# Hematopoietic anomalies fuels multiple sclerosis

**DOI:** 10.1093/lifemedi/lnac019

**Published:** 2022-07-01

**Authors:** Zhongyang Wu, Xu Zhou

**Affiliations:** Division of Gastroenterology, Hepatology and Nutrition, Department of Pediatrics, Boston Children’s Hospital and Harvard Medical School, Boston, MA 02115, USA; The Broad Institute of MIT and Harvard, Cambridge, MA 02142, USA; Division of Gastroenterology, Hepatology and Nutrition, Department of Pediatrics, Boston Children’s Hospital and Harvard Medical School, Boston, MA 02115, USA; The Broad Institute of MIT and Harvard, Cambridge, MA 02142, USA

## Abstract

**Graphical Abstract:**
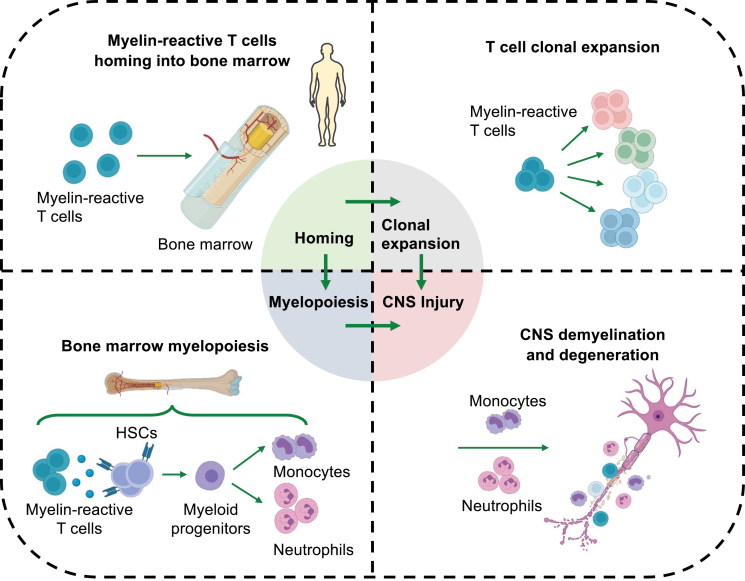

Multiple sclerosis (MS) is an autoimmune inflammatory disease with a hallmark of chronic demyelinating lesions in the center nervous system (CNS). Although MS is deemed as a white matter disease, MS lesions also occur in brain gray matter, spinal cord, and optical nerve. These lesions are mainly characterized by massive infiltration of T lymphocytes, plasma cells, myeloid cells (i.e. monocytes and neutrophils) as well as the activation of resident glial cell types [1]. Failure to resolve MS lesions within few months following their occurrence often leads to progressive demyelination and axon degeneration [1,2]. Although substantial progress has been made in earlier diagnosis and disease-modifying therapies, unmet clinical need persists for MS patients with all forms of progressive disease [2,3]. This has led to renewed research interests to understand the pathological machinery underlying disease progression.

MS pathology is thought be primarily driven by autoreactive T cells. Recently, a surge of circulating monocytes and neutrophils is observed to be associated with MS disease progression, linking myeloid cell types to disease pathology [4,5]. The underlying immune basis and clinical relevance of these findings in MS patients remain to be identified. In addition, given the short lifespan of myeloid cell types (within days) and their massive infiltration into the CNS, supply of these cells would rely on continuous bone marrow hematopoiesis. Yet, the dynamic activity of hematopoietic differentiation within the bone marrow niche and its potential impact on MS progression are largely unknown.

In a recent issue of *Cell*, Shi and colleagues provide a comprehensive view of bone marrow hematopoietic stem and progenitor cells (HSPC) in MS at single-cell resolution and revealed a previously unrecognized role of myelopoiesis in disease progression [6]. To characterize the activity of bone marrow hematopoietic system, the authors performed single-cell sequencing to analyze bone marrow HSPCs and their downstream cellular lineages in active MS patients. A snapshot of single-cell transcriptome in MS patients revealed a continuum of hematopoietic differentiation trajectories biased toward myeloid cell lineages. This was concomitant with an increased output of myeloid cells as well as clonal expansion of T cells in MS patients [6]. Shi and colleagues further demonstrated that these findings in patients were mirrored in the mouse model of experimental autoimmune encephalomyelitis (EAE), an animal model of MS. To spatially and temporally track HSPC-derived downstream cell types during disease progression, the authors conducted lineage tracing of HSPCs using an Fgd5-CreER/tdTomato mouse line. They found that an early increase of HSCs and myeloid progenitor cells in EAE mice are responsible for the increased bone marrow output of inflammatory monocytes and neutrophils that migrated into blood, spleen, brain, and spinal cord tissues [6]. Next, using a TCR transgenic line that harbor myelin-reactive T cells (2D2 mice), the authors identified CXCR4+ myelin-reactive T cell as the predominant T-cell population that migrates into the bone marrow. This process depends on chemokine CXCL12 that was likely derived from bone marrow hematopoietic niche [7]. Once arriving at bone marrow, these myelin-reactive T cells produce CCL5 to activate CCR5-expressing HSCs, leading to augmented myelopoiesis and T-cell clonal expansion [6]. Subsequently, pharmacological or genetic disruption of CCL5-CCR5 axis ablated the enhanced myelopoiesis, CNS inflammation, and demyelination [6]. Thus, these findings demonstrate an importance and unexpected role of the bone marrow, a rather distal hematopoietic organ, to the inflammation and autoimmune in the central nerve system (Fig. 1). Targeting tissue processes that are outside of the blood–brain barrier provides an attractive treatment option to control MS.

**Figure 1.**
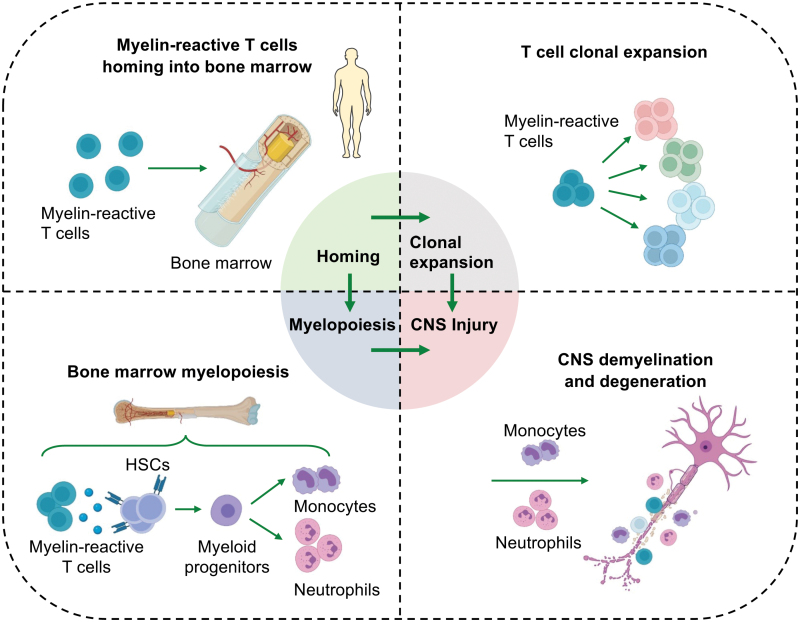
Mechanisms of aberrant bone marrow myelopoiesis in multiple sclerosis. In multiple sclerosis (MS), peripheral myelin-reactive T cells preferentially migrate into bone marrow niche that produce C-X-C motif chemokine ligand 12 (CXCL12) to attract myelin-reactive T cells. Once arrive the bone marrow, myelin-reactive T cells produce C-C motif chemokine ligand 5 (CCL5) to stimulate C-C chemokine receptor 5 (CCR5)-expressing HSCs (hematopoietic stem cells), leading to augmented output of monocytes and neutrophils. These newly generated myeloid cells can either facilitate the clonal expansion of myelin-reactive T cells or infiltrate into CNS to accelerate demyelination and degeneration.

The comprehensive analysis of the bone marrow response to active MS by Shi and colleagues also shed light on the ontogeny of nearly all immune cell types involved in MS immunopathology. Autologous hematopoietic stem cell transplantation (AHSCT) has been increasingly explored as an alternative treatment for drug-resistant progressive MS [8,9]. The goal of this treatment is to eliminate autoreactive T and B cells by “reset” the immune system with “brand new” lymphoid and myeloid systems. A real-world cohort analysis revealed that MS patients receiving AHSCT had no new MRI lesions in 90% of participants at 2 years, and in 85% of the patients by 4 years [9]. These clinical findings further support a central role of the bone marrow compartment in driving CNS inflammation. Accordingly, Shi and colleagues argued that future investigations can be directed at identifying effective approaches to precisely modulate bone marrow immunity. The success of these new approaches may benefit MS patients by avoiding chemotherapy or radiation therapy entirely. The single-cell RNA-sequencing dataset of MS patient cohorts from this work serve as a valuable resource to identify the hematopoietic origin of MS immunopathogenesis and progression.

In addition to bone marrow, bone tissues surrounding CNS, such as the skull, can support a reservoir of immune cells that survey the CNS meningeal and parenchyma tissues [10]. Shi and colleagues showed that HSPCs were activated both within the CNS-surrounding bone marrow and remote femur bone marrow in EAE mice [6], although the relative contributions to CNS demyelination from different bone marrow compartments remain to be defined. Notably, the augmented myelopoiesis and biased TCR Vβ usage was also observed in the CNS-surrounding bone marrow during EAE [6]. This biased TCR Vβ usage may be explained by an increased T-cell diversity driven by activated myeloid cells within bone marrow. Determining TCR repertoire and characterizing antigen presentation of bone marrow myeloid cells in the EAE model may provide a clue to the mechanisms of altered T-cell diversity.

Shi *et al.* illustrated an intriguing link between altered HSPC lineage differentiation and disease activity in MS patients. Could the aberrant HSPC activity serve as a biomarker to predict the risk of MS progression, relapse or responsiveness to immune therapy? Are such changes in the bone marrow relatively transient or long-lasting? Further investigations of the association between increased HSPC activity and evolution of MS disease activity will provide answers. Additional studies of both intrinsic risk factors and extrinsic environmental influence that led to the dynamic alterations of HSPC may reveal novel targets for a new generation of treatment strategy that modulates MS disease by manipulating immune systems and inflammatory response outside of brain.
